# Acceptability and effectiveness of a web-based psychosocial intervention among criminal justice involved adults

**DOI:** 10.1186/s40352-017-0048-z

**Published:** 2017-03-11

**Authors:** J. D. Lee, B. Tofighi, R. McDonald, A. Campbell, M. C. Hu, E. Nunes

**Affiliations:** 1 New York University School of Medicine, Department of Population Health, 227 E. 30th St., 10016 New York, NY USA; 2 New York State Psychiatric Institute, Department of Psychiatry, Columbia University College of Physicians and Surgeons, New York, NY USA; 3 Department of Psychiatry, Columbia University College of Physicians and Surgeons, New York, NY USA

**Keywords:** Criminal justice, Web-based treatment, Addiction, Neurocognitive performance

## Abstract

**Background:**

The acceptability, feasibility and effectiveness of web-based interventions among criminal justice involved populations are understudied. This study is a secondary analysis of baseline characteristics associated with criminal justice system (CJS) status as treatment outcome moderators among participants enrolling in a large randomized trial of a web-based psychosocial intervention (Therapeutic Education System [TES]) as part of outpatient addiction treatment.

**Methods:**

We compared demographic and clinical characteristics, TES participation rates, and the trial’s two co-primary outcomes, end of treatment abstinence and treatment retention, by self-reported CJS status at baseline: 1) CJS-mandated to community treatment (CJS-mandated), 2) CJS-recommended to treatment (CJS-recommended), 3) no CJS treatment mandate (CJS-none).

**Results:**

CJS-mandated (*n* = 107) and CJS-recommended (*n* = 69) participants differed from CJS-none (*n* = 331) at baseline: CJS-mandated were significantly more likely to be male, uninsured, report cannabis as the primary drug problem, report fewer days of drug use at baseline, screen negative for depression, and score lower for psychological distress and higher on physical health status; CJS-recommended were younger, more likely single, less likely to report no regular Internet use, and to report cannabis as the primary drug problem. Both CJS-involved (CJS -recommended and -mandated) groups were more likely to have been recently incarcerated. Among participants randomized to the TES arm, module completion was similar across the CJS subgroups. A three-way interaction of treatment, baseline abstinence and CJS status showed no associations with the study’s primary abstinence outcome.

**Conclusions:**

Overall, CJS-involved participants in this study tended to be young, male, and in treatment for a primary cannabis problem. The feasibility and effectiveness of the web-based psychosocial intervention, TES, did not vary by CJS-mandated or CJS-recommended participants compared to CJS-none. Web-based counseling interventions may be effective interventions as US public safety policies begin to emphasize supervised community drug treatment over incarceration.

## Background

The US criminal justice system (CJS) population is composed primarily of adults with substance use disorders, chiefly through arrests and convictions for activities to sustain illicit drug use or for offenses committed under the influence of drugs and alcohol (Carson and Golinelli, 2013; Feucht and Gfroerer, 2011; Field, 1989). Incarcerated populations are more than 13 times more likely to be dependent on drugs than the general population (Fazel et al., 2006), and will likely struggle with substance use disorders upon community re-entry. Community drug treatment interventions, including pre-trial diversion, community supervision, and problem-solving court programs (e.g. drug courts, mental health courts), are increasingly being implemented as cost-saving and evidence-based public health alternatives to incarceration (Feucht and Gfroerer, 2011). U.S. sentencing trends and national guidelines have been prioritizing drug treatment over incarceration for non-violent drug related offenses (ONDCP, 2013).

Once in community treatment, CJS-involved patients may have unique characteristics and prognoses. CJS vs. non-CJS treatment populations are disproportionately poor, unemployed, have lower literacy rates (Greenberg et al., 2007; Hudson, 1987), and have worse mortality and general health outcomes than non-CJS age-matched populations (Binswanger et al., 2007; Greenberg et al., 2007). The outcome data for mandated versus voluntary substance abuse treatment in the criminal justice system is mixed. Mandated, compared to voluntary treatment may compel unmotivated or even hostile participants into unwanted treatment, making positive treatment outcomes less likely even if retention rates are better (Perron and Bright, 2008). Other studies have shown that mandated treatment outcomes are generally identical and often better compared to voluntary treatment in CJS-involved populations (Farabee et al., 1998; NIDA, 2014; Taxman, 1998). Further studies of CJS-involved individuals in community drug treatment programs have revealed mixed outcomes compared to non-CJS treatment populations (Farabee et al., 1998; Lee et al., 2012). A recent study of male probationers demonstrated greater unmet needs for substance abuse treatment compared to non-CJS substance abusers (Feucht and Gfroerer, 2011).

Novel community addiction treatment interventions, including health technology-based interventions, may have variable effects depending on patient sub-populations and CJS status. An important area of health technology innovation is web- or Internet-based drug and alcohol counseling. Compared to traditional face-to-face counseling, web-based or other mobile health (‘mHealth’) psychosocial interventions may be more scalable as a result of cost efficiencies and accessibility (Carroll and Rounsaville, 2010; Marsch and Dallery, 2012). Few studies have assessed computer and Internet literacy and the feasibility of these interventions in CJS populations (Alemagno et al., 2009; Chaple et al., 2013; Ford and Vitelli, 1992; Walters et al., 2014). Even fewer have been conducted amongst community-dwelling CJS-involved treatment populations with real-time Internet access, as opposed to non-Internet, computer-based modules (Alemagno et al., 2009; Walters et al., 2014). To our knowledge, only one web-based substance use intervention specifically for CJS-involved persons is in development (Walters et al., 2014).

The objective of this secondary data analysis was to compare baseline demographics and clinical characteristics and treatment outcomes of CJS-involved participants to participants with no apparent CJS involvement entering a national multisite web-based psychosocial addiction treatment study (WEB-TX) (Bickel et al., 2008; Campbell et al., 2012). This study was funded and conducted by NIDA's Clinical Trials Network (NIDA CTN). WEB-TX had broad inclusion and few exclusion criteria and was intended to draw a diverse sample of patients seeking treatment for drug or alcohol problems in community-based treatment programs in the United States. Based on previously described disadvantages among CJS-involved populations, including lower educational achievement, employment, and computer literacy, we hypothesized CJS-involved participants would have more severe addiction disorders, more psychiatric co-morbidities, and report less Internet use at baseline compared to participants with no reported CJS involvement. Similarly, we hypothesized CJS-involved participants would have poorer treatment outcomes overall, including less web-based intervention exposure, and lower treatment retention and end of study abstinence.

## Methods

### Setting and participants

Full details about the study have been described previously (Campbell et al., 2012). Briefly, the impact of a web-based version (Bickel et al., 2008) of the Community Reinforcement Approach (Hunt and Azrin, 1973), including prize-based contingency management (Budney et al., 1991), was assessed among 507 participants enrolled in 10 outpatient substance abuse treatment programs located across the United States. Potential participants were informed about the opportunity to participate in the trial at the time they entered one of the collaborating treatment programs. Eligible participants were in the first 30 days of the current treatment episode, reported using an illicit substance within the 30 days prior to screening, and were not receiving opioid replacement medications such as methadone or buprenorphine. Of the 1,781 participants screened, 848 were ineligible (47.6%), 426 (23.9%) were eligible but did not enroll in the study, and 507 (28.5%) were eventually randomized. The primary reasons for ineligibility were: no illicit substance use in the 30 days prior to screening (83.5%); not planning a treatment episode of at least 90 days (11.8%); in the current treatment episode greater than 30 days (8.5%); and currently prescribed an opioid replacement therapy (7.9%).

### Study design and intervention

Enrolled participants were randomly assigned to 12 weeks of: (1) treatment-as-usual (TAU); or (2) modified TAU + Therapeutic Education System (TES), whereby TES substituted for approximately 2 h of usual care (i.e., clinician-delivered groups). All participants were asked to provide self-reported substance use and urine drug and breath alcohol screens twice per week during the treatment phase. TES (Bickel et al., 2008; Campbell et al., 2014) includes 62 web-delivered multimedia modules or topics which focus on cognitive behavioral relapse prevention skills, psychosocial functioning, and HIV and other sexually transmitted infection prevention and treatment information. Participants were asked to complete at least 4 modules per week; that is, 48 of the 62 modules over the 12 week treatment phase. The contingency management component of TES is a prize-based incentive system (Petry et al., 2005; Stitzer et al., 2010) whereby participants can earn intermittent prizes for submitting negative urine/breath alcohol screens and completing TES modules (up to 4 per week).

### Measures

Demographic variables included sex, age, race/ethncity, education, marital status, employment status, criminal justice drug treatment status, and lifetime incarceration and criminal behavior history. Criminal justice status was categorized into 3 subgroups per a single self-reported item about whether participants were mandated or recommended for treatment by CJS authorities: CJS-mandated, CJS-recommended, or CJS-none. Frequency of Internet use was a self-reported categorical measure, inquiring how often participants accessed the Internet in the past 30 days.

Screening for major depressive disorder, generalized anxiety disorder and panic disorder was completed using the Patient Health Questionnaire (PHQ) (Spitzer et al., 1999; Spitzer et al., 2006). The Brief Symptom Inventory-18 (Derogatis, 2000) (total global score) measured overall psychological distress level. Primary substance of abuse and age of onset of substance use dependence were both collected using the DSM-IV symptom questionnaire (Hudziak et al., 1993). The Coping Strategies Scale (Litt et al., 2003; Litt et al., 2012) (total number of endorsed strategies (0–23); endorsed strategies are those used occasionally or frequently) assessed change processes used in altering substance use behavior. The Euro Quality of Life Scale-EQ5D (EuroQol Group, 1990) assessed participants’ perception of their physical health using a visual analogue scale (range 0–100; where 100 is the best health). Medical service utilization was measured for the 30 days prior to initial enrollment into the study and included number of doctor visits, emergency department visits, and hospital admissions.

The MicroCog computerized Assessment of Cognitive Functioning has been normalized and standardized for adults (Powell et al., 1993). Based on our previous work (Aharonovich et al., 2008), a custom version of the MicroCog (20–25 min in length) was used to measure working memory (Numbers Forward and Reversed), immediate/delayed memory (Wordlist 1 and 2), logical association of familiar concepts (Analogies), and spatial recognition/logic (Object Match A and B; Clocks). Subtest raw scores were transformed to scaled scores (μ = 10.0, σ = 3.0) and descriptively defined (Powell et al., 1993) as: μ ≤ 4 (below average), 8 > μ > 4 (low average), 13 > μ ≥ 8 (average), and μ ≥ 13 (above average).

Acceptability of the TES intervention was measured on a 0–10 point scale (higher scores corresponding to greater acceptability) using 5 indicators: how interesting, how useful, quantity of new information, how easy to understand, and how satisfied. Participants completed the measure at the end of the 12-week treatment phase.

Abstinence from drugs and alcohol was evaluated twice weekly during the 12-week treatment phase. Abstinence was defined as a negative urine toxicology test for 10 drugs of abuse and self-reports indicating no drug or alcohol use measured using the Timeline Follow Back method (Sobell and Sobell, 1992). Abstinence data was considered missing if the urine screen was missing or if the urine screen was negative and the self-report was missing. The outcome was a binary measure of abstinence (yes or no) throughout the last 4 weeks of treatment (i.e., weeks 9–12).

### Statistical analysis

Either the *χ* ^2^ test, F-test, or the Kruskal-Wallis test (non-parametric equivalent of the one-way independent ANOVA) was used to compare CJS-mandated and CJS-recommended with CJS-none across baseline variables of interest. A p-value of < .05 was considered significant. Neurocognitive testing scores were adjusted for age, education, and baseline abstinence status.

The study’s primary outcomes, abstinence throughout the final four weeks of treatment and retention at week 12 (dichotomous, yes or no), were analyzed using generalized linear mixed effect models (with logit link conducted with Proc GLIMMIX in SAS). The generalized linear mixed model is used to handle correlated data from repeated measurements on categorical outcomes and provides robust inference with respect to misspecification of within-subject correlation (Breslow and Clayton, 1993). Models included treatment arm (TES vs. TAU), CJS subgroup, and abstinence at baseline and site and subject were treated as random effects. Interactions between treatment, CJS subgroup, and abstinence at baseline were tested and included in the final model if significant at *p <* .05. Time was included in the model testing abstinence (*n* = 469); 38 cases were removed that were missing all four weeks of data. The correlation between the repeated measurements within-subject was modeled using the first-order auto regressive structure. Missing data was assumed missing at random and this differential attrition did not impact analysis of the primary outcome. All analyses were conducted using SAS version 9.3.

## Results

### Demographic, internet use, substance use, general health baseline characteristics

Table 1 presents baseline demographic features of the randomized sample. Overall, the two CJS-involved groups were more likely to be male, younger, and using cannabis as a primary drug versus opioids. CJS-mandated compared to CJS-none were significantly more likely to be male, uninsured, report more cannabis, alcohol, and stimulants as the primary drug problem, report fewer days of drug use at baseline, screen negative for depression, and score lower for psychological distress and higher on physical health status; CJS-recommended compared to CJS-none were younger, more likely single, to report cannabis as the primary drug problem, and have a lower proportion reporting daily Internet use (35%).

**Table 1:** Baseline demographic and clinical characteristics of the randomized sample (*N* = 507) as a function of criminal justice system (CJS) involvement

Demographics/Clinical Characteristics	CJS-None (*n* = 331)	CJS-Recommended (*n* = 69)	CJS-Mandated (*n* = 107)	Significance Between Groups
	Mean (SD) or %			F or χ^2^. *P*-value
Age	36.08 (10.80) ^a^	30.93 (10.90) ^b^	33.78 (10.54) ^ab^	*F* (2, 504) = 7.28, *p* < .001
Female	41.82^a^	31.88^ab^	29.91^b^	*x* ^2^ (2) = 6.12, *p* = .047
Race/Ethnicity				*x* ^2^ (6) = 6.56, *p* = .36
White (ref.)	55.89	52.17	42.99	
Black	21.45	21.74	24.30	
Hispanic/Latino	10.27	10.14	13.08	
Other/Mixed	12.39	15.94	19.63	
Education				*x* ^2^ (4) = 4.05, *p* = .40
< High School	21.45	30.43	24.30	
High School	61.33	56.52	63.55	
> High School	17.22	13.04	12.15	
Employed	41.99	40.58	39.25	*x* ^2^ (2) = 0.26, *p* = .88
Single/Never Married	56.80^a^	73.91^b^	64.49^ab^	*x* ^2^ (2) = 7.81, *p* = .02
Internet Use (90d)				*x* ^2^ (6) = 14.60, *p* = .02
None (no)	27.49	21.74	25.23	
Less than once/week	7.25	10.14	5.61	
At least once/week	15.41	33.33	20.56	
At least once/day (ref)	49.85	34.78	48.60	
Insurance (90d)	78.25^a^	73.91^ab^	65.09^b^	*x* ^2^ (2) = 7.43, *p* = .02
Baseline drug abstinence	53.47	53.62	57.01	*x* ^2^ (2) = 0.42, *p* = .81
Days of any Substance Use (90d)	47.47 (27.06) ^a^	44.29 (27.13) ^a^	36.02 (25.54) ^b^	*x* ^2^ (2) = 14.70^+^, *p* < .001
Age of Onset (Dependence)	22.82 (9.00)	20.38 (7.78)	21.47 (6.73)	*F* (2, 460) = 2.29, *p* = .11
Years of Substance Use (Dependence)	13.22 (10.66)	10.67 (8.90)	12.83 (10.06)	*x* ^2^ (2) = 2.27^+^, *p* = .32
Primary Substance				*x* ^2^ (10) = 24.12, *p* = .007
Alcohol	20.54	13.04	25.23	
Cocaine	22.05	24.64	11.21	
Other Stimulants	12.08	11.59	19.63	
Opioids (ref.)	24.47	20.29	12.15	
Cannabis	18.73	28.99	29.91	
Other	2.11	1.45	1.87	
Current Depressive Disorder	24.17^a^	17.39^ab^	13.08^b^	*x* ^2^ (2) = 6.61, *p* = .04
Current Generalized Anxiety Disorder	29.61	23.19	24.30	*x* ^2^ (2) = 1.92, *p* = .38
Current Panic Disorder	17.52	23.19.20	16.82	*x* ^2^ (2) = 1.39, *p* = .50
Psychological Distress (Brief Symptom Inventory)	14.38 (12.62) ^a^	12.49 (12.31) ^ab^	11.51 (12.37) ^b^	*x* ^2^ (2) = 8.87^+^, *p* = .02
Physical Health (0–100)	72.24 (19.67) ^a^	70.03 (21.68) ^a^	76.85 (17.78) ^b^	*x* ^2^ (2) = 6.17^+^, *p* = .046
Social Adjustment Total Score	2.21 (0.48)	2.17 (0.54)	2.09 (0.50)	*F* (2, 504) = 1.71, *p* = .18

^a-b^ Different superscripts indicate statistical significance at *p* < .05 for pairwise comparisons between two criminal justice groups

+ Non-parametric Kruskal-Wallis tests were used

### Recent incarceration and self-reported criminal activity

The mean days of self-reported criminal activity obtained at baseline were similar across CJS subgroups; on average 4–6 days of the last 90 days were characterized by criminal behavior, the most common of which were directly drug-related (sale, possession) or driving while intoxicated [Table 2]. As expected based on self-reported CJS treatment mandates, CJS-mandated and CJS-recommended reported significantly higher rates of recent incarceration (35% and 39% vs. 13%, *p* < 0.001).

**Table 2:** Baseline (last 90 day) rates of incarceration and criminal activity by criminal justice status group

	CJS- None (*n* = 331)	CJS-Recommended (*n* = 69)	CJS- Mandated (*n* = 107)
	Mean (SD) or %		
Days detained/incarcerated % participants detained/incarcerated	1.53 (8.45) 12.99^a^	5.88 (12.19) 39.13^b^	5.08 (12.36) 35.51^b^
Days of criminal activity	4.94 (16.08)	5.14 (15.83)	4.41 (16.69)
% Committed, Charged or Convicted			
Drug dealing or drug charges	15.41	31.88	18.69
Shoplifting/theft/auto	14.0	15.94	9.35
Robbery/burglary	2.11	10.14	2.80
Aggravated assault	4.83	5.80	2.80
Sexual assault	0.00	0.00	0.00
DUI	19.94	27.54	20.56
Other	6.95	24.64	19.63

a-b. Difference in % participants detained/incarcerated, *p* < 0.001

### Neurocognitive data

Table 3 presents baseline neurocognitive data for the scaled scores on the MicroCog (Drozdick et al., 2004) subtests. Scores were age and education-adjusted. The CJS -mandated and –recommended groups performed significantly better on subtests of working memory (Numbers Reversed) compared to CJS-none. Other subtests, such as attention and concentration (Numbers Forward), reasoning, cognitive flexibility and spatial processing did not differ significantly.

**Table 3:** Baseline Age/Education-Adjusted Neurocognitive Data (*N* = 507)

MicroCog Subtest Scaled Scores	Illegal Behavior/Criminal Justice Involvement			Significance Between Groups^a^
	CJS-none (*n* = 331)	CJS-Recommended (*n* = 69)	CJ Mandated (*n* = 176)	
	Mean (SD)			F-test, *p* value
Numbers Forward Total Score	8.41 (2.70)	8.94 (2.56)	8.94 (2.85)	F (2, 503) = 2.49, *p* = .08
Numbers Reversed Total Score	7.90 (2.51)	8.88 (3.08)	8.41 (2.87)	F (2, 503) = 4.60, *p* = .01^b^
Wordlist 1 Total Score	7.40 (4.59)	7.32 (4.58)	7.91 (4.18)	F (2, 502) = 0.54, *p* = .59
Wordlist 2 Total Score	9.62 (3.17)	9.59 (3.33)	9.73 (3.29)	F (2, 502) = 0.04, *p* = .96
Analogies Total Score	6.71 (2.90)	6.48 (2.57)	6.79 (2.92)	F (2, 503) = 0.28, *p* = .75
Object Match A Total Score	8.30 (3.86)	8.42 (4.31)	8.08 (3.82)	F (2, 499) = 0.19, *p* = .83
Object Match B Total Score	8.50 (3.16)	8.62 (3.37)	8.88 (2.95)	F (2, 498) = 0.60, *p* = .55
Clocks Total Score	11.00 (1.85)	10.77 (2.04)	11.16 (1.71)	F (2, 503) = 0.94, *p* = .39

^a^ adjusted for baseline abstinence

^b^. Numbers reversed total scores is lower in CJS-none than CJS-recommended group. (t = 2.79, *p* < .01)

### TES module completion and acceptability

Among participants randomized to the TES arm, module completion was similar across the CJS subgroups. Total modules completed were M = 37.2 (SD = 16.7) for the non-CJS subgroup, M = 36.2 (SD = 17.1) for the CJS-recommended subgroup and M = 36.2 (SD = 17.3) for the CJS-mandated subgroup (out of a recommended 48 modules or 4 modules per week for 12 weeks). Acceptability of TES (on a scale of 0–10, with 10 corresponding to higher acceptability) was high among all CJS subgroups: M = 8.25 (SD = 1.56), M = 8.40 (SD = 1.76), and M = 8.33 (SD = 1.51) among none-CJS, CJS-recommended, and CJS-mandated subgroups, respectively.

### Abstinence during the last four treatment weeks and treatment retention

The primary outcome of the randomized trial was drug and alcohol abstinence in the final four weeks of treatment. Final four-week abstinence was tested as a function of treatment arm, baseline abstinence (i.e., baseline urine drug and breath alcohol negative), CJS status, and time (Table 4). The three-way interaction of treatment, baseline abstinence and CJS status was not significant (*p* = 0.41). Per the trial’s main results, there was a two-way interaction between treatment arm and baseline abstinence (*p* = .080) whereby among participants not abstinent at baseline, the TES treatment effect, compared to treatment-as-usual, was significant (*p* = .003). There were no significant effects of TES, regardless of baseline abstinence status, within the three CJS subgroups.

**Table 4:** Generalized Linear Model for Abstinence in the Final Four Weeks of Treatment by Treatment Arm, Baseline Abstinence, CJS Status, and Time

	F-test	p-value
Time	F (1, 2450) = 0.13	.717
Baseline Abstinence	F (1, 2450) = 58.28	<.001
Treatment Arm (TES vs. TAU)	F (1, 2450) = 6.98	.008
CJS Status (None, Recommended, Mandated)	F (2, 2450) = 1.75	.174
Treatment x Baseline Abstinence	F (1, 2450) = 3.07	.080

Retention in the outpatient treatment program at week 12 was tested as a function of treatment arm, baseline abstinence, and CJS status. The three-way and two-way interactions were not significant. Treatment retention by CJS subgroup neared significance but did not meet the *p* < .05 threshold (*p* = .06): CJS-mandated (50.5%), CJS-recommended (52.2%), and CJS-none (39.9%). After controlling for treatment arm and baseline abstinence in the final model, differences in treatment retention by CJS subgroup (*p* = .169) were not significant. Also in the final model, baseline abstinence (*p* = .013) and treatment (*p* = .069, a trend level of significance) were associated with greater retention

## Discussion

This analysis of self-reported baseline criminal justice status upon entry into a randomized controlled trial of web-based addiction treatment indicated several notable findings: compared to participants with no reported CJS involvement, those mandated or recommended to treatment by CJS authorities were younger, more likely to be male, and more cannabis-involved. Regardless of CJS-status, all participants experienced the trial or the TES web-based psychosocial intervention in a similar fashion; there were no differences observed in the rate of TES participation, retention in treatment, or end-of-study drug and alcohol abstinence. These findings demonstrate a high level of feasibility and acceptability of web-based interventions among community-based addiction treatment attendees, including those that are involved to varying degrees with the CJS system.

While CJS-involved populations may have more unmet treatment needs (Fazel et al., 2006; Feucht and Gfroerer, 2011), our findings generally were contrary to our hypotheses that criminal justice involved individuals would show greater levels of impairment and less success in web-based treatment; there were no differences in terms of TES initiation, completion, or the trial’s main outcomes of retention and abstinence across the three CJS subgroups. Our hypotheses were based on prior studies characterizing CJS populations as disproportionately poor and unemployed, with low literacy rates and worse mortality and general health outcomes (Binswanger et al., 2007; Greenberg et al., 2007; Hudson, 1987). Additionally, we considered prior studies suggesting that mandated treatment outcomes were often worse compared to other populations (Perron and Bright, 2008; Simpson et al., 1997; Taxman, 1998).

The sample as a whole showed impairment (e.g. relatively low educational attainment, low employment, and relatively higher rates of anxiety and depression), neurocognitive tests below population norms in general, and increasingly lower neurocognitive scores as tests increased in difficulty (e.g. from Numbers Forward to Numbers Reversed). Due to an assumption of potentially lower socioeconomic status and educational achievement, we expected lower rates of Internet use and lower neurocognitive ratings among CJS-involved participants, neither of which were found. If anything, the data suggest that within this relatively impaired overall sample of treatment seekers, those with criminal justice involvement may have less impairment, and be more ready to benefit from a computer-delivered intervention. The younger age of the criminal justice involved patients might explain some of these results, such as a higher overall proportion reporting some recent Internet use. Younger addiction treatment entrants are generally more likely to use the Internet (Cohall et al., 2011), which may have applied to CJS-involved participants in this study. Further, because the CJS-involved participants in this study were able to seek outpatient drug treatment, as opposed to serving jail time, it may be that their criminal histories were shorter and less involved (e.g., infractions related to driving under the influence or possession of cannabis). This would also account for the small number of differences found between the CJS subgroups.

Other studies have found computer-delivered interventions to be successful with CJS involved patients. Inmates completing an attitudinal survey were highly receptive towards receiving computerized adjunct psychotherapy and were “equally divided” between receiving computerized versus clinician-delivered therapy sessions (Ford and Vitelli, 1992). Participants agreed that the computer sessions were “a good use of time”, “enjoyable”, and “interesting” (Ford and Vitelli, 1992). A randomized trial (Alemagno et al., 2009) examined the effectiveness of a brief computerized motivational intervention compared to usual education activities, assessing whether it could decrease HIV risk-behaviors and increase HIV testing among a community-supervised population (i.e. probationers). At 2-months post intervention, the number of those who obtained HIV testing were significantly higher in the experimental vs. control group. Furthermore, those randomized to the computerized intervention appeared to have increased positive attitudes towards changing their risk and awareness of HIV (Alemagno et al., 2009). In a recent clinical trial using TES in 10 state prisons, results showed high rates of TES module completion, improvements in copings skills, and a “more favorable view” of TES than of standard care, which consisted of 2 h, weekly group sessions with a certified Addictions Counselor (Chaple et al., 2013). Although this research is promising, Internet-delivered treatment has been largely unexplored in a community setting, under everyday pressures, although studies are underway (Walters et al., 2014). The current study is a step to begin filling this knowledge gap by exploring computer literacy and web-based treatment among community-based, CJS-involved individuals with substance use disorders.

This analysis has several strengths, including comprehensive assessment of baseline patient characteristics, and a large sample drawn from a wide variety of outpatient clinical treatment programs across the United States. There are also clear limitations. First, since participants were selected from outpatient programs, these findings may not be generalizable to other populations, such as inpatient or incarcerated populations. Second, since eligibility criteria required at least some recent (past 30 days) illicit substance use, participants with alcohol use disorders only and no other drug use were not included. Third, our assessment of medical and psychiatric morbidity was limited, only relying on self-report. A more comprehensive medical and psychiatric assessment with collateral history from participants’ families or their primary care physicians might have yielded different findings. Fourth, our categorization of CJS status was gathered from a single baseline questionnaire item, was not confirmed with administrative CJS data, and may contribute to a loss of information about individual differences (Altman and Royston, 2006). Finally, we do not know if the neurocognitive data reflect premorbid capacity or substance-induced cognitive changes, which could have been further teased out with collateral history.

## Conclusions

In summary, this secondary analysis reveals that CJS participants entering outpatient addiction treatment are more likely young, male, and cannabis-involved. Baseline Internet use, web-based treatment uptake, and end-of-study abstinence and retention outcomes did not vary significantly by CJS status, implying CJS-involved participants are as likely to use a web-based psychosocial intervention and achieve similar outcomes as general patient populations. We detected no differences in the TES intervention’s effects by the three CJS classes, nor did these sub-groups confound an earlier overall finding of baseline abstinence predicting treatment retention. CJS-involved participants appeared to experience the usual expected benefits in this large multisite randomized trial. These results should encourage treatment providers, policy makers, and CJS authorities to further consider online psychosocial interventions as viable and appropriate therapeutic approaches in CJS addiction treatment populations.
